# Evidence of Potential Anammox Activities from Rice Paddy Soils in Microaerobic and Anaerobic Conditions

**DOI:** 10.3390/biology13070548

**Published:** 2024-07-19

**Authors:** Anamika Khanal, Hyung-Geun Song, Yu-Sung Cho, Seo-Yeon Yang, Won-Seok Kim, Alpana Joshi, Jiho Min, Ji-Hoon Lee

**Affiliations:** 1 Department of Agricultural Chemistry, Jeonbuk National University, Jeonju 54896, Republic of Korea; anamika.khanal@jbnu.ac.kr (A.K.); 201515780@jbnu.ac.kr (H.-G.S.); dbtjd0701@jbnu.ac.kr (Y.-S.C.); dustj@jbnu.ac.kr (S.-Y.Y.); 2 Division of Advanced Nuclear Engineering, Pohang University of Science and Technology, Pohang 37673, Republic of Korea; wkim23@postech.ac.kr; 3 Department of Agriculture Technology & Agri-Informatics, Shobhit Institute of Engineering & Technology, Meerut 250110, India; alpana.joshi@shobhituniversity.ac.in; 4 Department of Bioenvironmental Chemistry, Jeonbuk National University, Jeonju 54896, Republic of Korea; 5 School of Chemical Engineering, Jeonbuk National University, Jeonju 54896, Republic of Korea; jihomin@jbnu.ac.kr; 6 Institute of Agricultural Science & Technology, Jeonbuk National University, Jeonju 54896, Republic of Korea

**Keywords:** anammox in paddy soil, bacterial community, anammox-specific gene, batch incubation

## Abstract

**Simple Summary:**

Anammox microorganisms are beneficial for use in wastewater treatment reactors. However, the application is limited due to the slow growth, high sensitivity, and the lack of a pure culture. To expand the sources of anammox bacteria, we used terrestrial soils from rice paddy fields to enrich and detect the anammox bacteria. This approach revealed potential anammox activities in both microaerobic and anaerobic soil conditions.

**Abstract:**

Anammox, a reaction in which microorganisms oxidize ammonia under anaerobic conditions, is used in the industry to remove ammonium from wastewater in an environmentally friendly manner. This process does not produce intermediate products such as nitrite or nitrate, which can act as secondary pollutants in soil and water environments. For industrial applications, anammox bacteria should be obtained from the environment and cultivated. Anammox bacteria generally exhibit a slow growth rate and may not produce a large number of cells due to their anaerobic metabolism. Additionally, their habitats appear to be limited to specific environments, such as oxidation-reduction transition zones. Consequently, most of the anammox bacteria that are used or studied originate from marine environments. In this study, anammox bacterial evidence was found in rice paddy soil and cultured under various conditions of aerobic, microaerobic, and anaerobic batch incubations to determine whether enrichment was possible. The anammox-specific gene (*hzs*A) and microbial community analyses were performed on the incubated soils. Although it was not easy to enrich anammox bacteria due to co-occurrence of denitrification and nitrification based on the chemistry data, potential existence of anammox bacteria was assumed in the terrestrial paddy soil environment. For potential industrial uses, anammox bacteria could be searched for in rice paddy soils by applying optimal enrichment conditions.

## 1. Introduction

Due to high nitrogen input to terrestrial and aquatic ecosystems from human activities such as the application of industrial nitrogen-based fertilizers, contamination with inorganic nitrogen compounds has been found and is an important issue in the agricultural and freshwater environments [1,2]. Highly accumulated inorganic nitrogen in the environment can be a serious pollution source and directly and/or indirectly influence human health [3,4]. For drinking water and wastewater treatments, nitrogen removal has been a central topic and microbial transformations of nitrogen compounds have been applied for the removal of inorganic nitrogen compounds [5,6,7]. Microorganisms transform nitrogen compounds via redox reactions including nitrification, denitrification, dissimilatory nitrate reduction to ammonia (DNRA), anaerobic ammonium oxidation (anammox), etc. [8,9,10]. In conventional wastewater treatment systems, the removal of nitrogen compounds is mainly achieved via microbial nitrification and denitrification processes. In general, ammonium in wastewater is oxidized to nitrite and nitrate by ammonium-oxidizing bacteria and nitrite-oxidizing bacteria. Then nitrate is anaerobically reduced to dinitrogen gas by denitrifying bacteria. Since the discovery of the anammox reaction [11], which is the anaerobic oxidation of ammonium and nitrite to dinitrogen gas, technologies using anammox reaction have been widely applied for nitrogen removal from wastewater treatment bioreactors [12,13,14]. For the full-scale bioreactors to treat wastewater using anammox reactions, enrichment or large-scale incubation of the bacterial cells would be one of the major issues, due to the known slow growth and high sensitivity of anammox bacteria [15,16,17]. Anammox microorganisms are highly sensitive to environmental factors and have a long doubling time of 2–3 weeks, resulting in an extended start-up period. These challenges hinder the large-scale application of anammox technology. The slow growth rate and high sensitivity of anammox bacteria, coupled with the absence of a pure culture, present significant obstacles to their efficient enrichment [18]. Consequently, developing effective techniques for enriching anammox bacteria is of considerable theoretical and practical importance [19].

Most cultures of anammox bacteria were enriched from sludges and marine origins using sequencing batch reactors (SBRs) [20]; however, anammox activities and molecular evidence have been repeatedly reported from terrestrial environments including rice paddy soils [21]. To expand the sources of anammox microorganisms for various applications, it is essential to explore enrichment possibilities from less-studied environmental media. In this study, we investigated suitable conditions for anammox enrichment from rice paddy soils. To simplify anammox enrichment using batch incubations without a sequencing batch reactor (SBR), we conducted several batch incubations and compared various conditions for anammox enrichment, focusing on the presence of oxygen, by incubating rice paddy soils in growth media with NO_2_^−^ and NH_4_^+^ under microaerobic and anaerobic conditions. Our goal was to develop effective anammox enrichment methods and compare three different experimental setups to identify the optimal conditions for enrichment using rice paddy sediments. Throughout the experiments, we monitored specific genes and microbial community structures over time to observe enhanced enrichment of anammox microorganisms in the three different incubation types. This study explored strategies to enrich and/or accelerate the activity of anammox bacteria using natural soil samples rather than activated sludges from wastewater treatment bioreactors.

## 2. Materials and Methods

### 2.1. Experimental Setups

#### 2.1.1. Experiment 1: Anaerobic Batch

**Figure 1:**
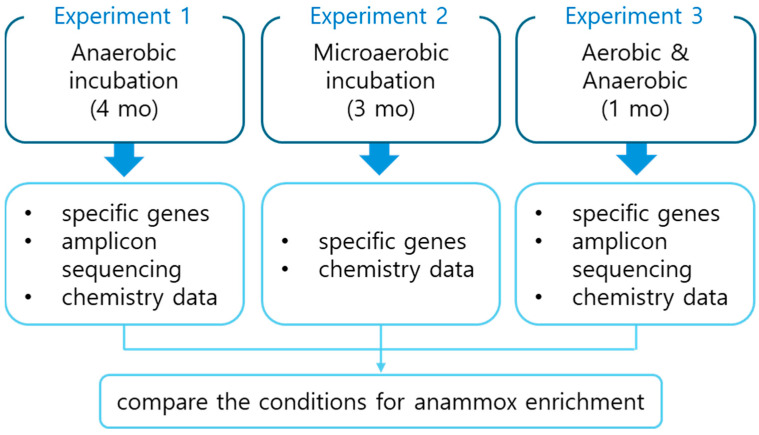
Flow chart of this study including the different incubation schemes.

Rice paddy soil (156 g) collected from approximately 20–30 cm depth of a local rice field (Figure S1A) was inoculated to 1 L of the anammox medium, composed of (per liter) 350 mg NaHCO_3_, 6 mg KH_2_PO_4_, 12 mg MgSO_4_·7H_2_O, 48 mg CaCl_2_·2H_2_O, 2 g HEPES, 69 mg NaNO_2_, 66.07 mg (NH_4_)_2_SO_4_, 1 mL trace element solution I, and 1 mL trace element solution II (Figure S1B). The trace element solution I was composed of (per L) 5 g EDTA and 5 g FeSO_4_·7H_2_O and the trace element solution II included (per L) 0.43 g ZnSO_4_·7H_2_O, 0.24 g CoCl_2_·6H_2_O, 0.99 g MnCl_2_·4H_2_O, 0.25 g CuSO_4_·5H_2_O, 0.22 g NaMoO_4_·2H_2_O, 0.19 g NiCl_2_·6H_2_O, 0.21 g Na_2_SeO_4_·10H_2_O, and 0.014 g H_3_BO_3_. The medium-containing 2-L Wheaton bottle was purged with 100% N_2_ gas for both the headspace and the solution at least for 30 min and was sealed with a chlorobutyl-isoprene rubber stopper. All the soil samples were sampled from the rice paddy field site, located inside the Rural Development Administration, Jeonju, South Korea (latitude 35°49′42.4″ N and longitude 127°02′38.4″ E; Figure S1A) [22]. The flow chart of this study is presented in Figure 1.

The slurry samples were analyzed for concentrations of nitrite, nitrate, and ammonium. Nitrate concentration was measured via the cadmium reduction method using Hach NitraVer 5 reagent (Loveland, CO, USA) at a wavelength of 400 nm and nitrite concentration was measured via the diazotization method using Hach NitriVer 3 reagent (Loveland, CO, USA) at 507 nm. Ammonium concentration was measured via the Nessler method using Hach Ammonia-Nitrogen reagent (Loveland, CO, USA) at 425 nm.

The gene copy number of the hydrazine synthase subunit A, *hzs*A gene, which is one of the central enzymes for anammox reactions [23], was monitored via quantitative PCR analysis using the Bio-Rad CFX Connect Real-Time System (Hercules, CA, USA) during the incubation period. A standard curve of *hzs*A gene was prepared for absolute amount gene copy number analysis. The DNA was inserted into a plasmid, quantified, and measured for generating a standard curve presenting the correlation between the Cq values and the number of gene copies. The primer set of hzsA_382F and hzsA_1857R used for this analysis is presented in Table S1.

Bacterial community structures of the soils were monitored using bacterial 16S rRNA gene amplicons analyzed via Illumina MiSeq (San Diego, CA, USA) along with the incubation times at 1 day, 15 days, and 56 days. Soil genomic DNA was extracted from 0.25 g soil for each sample using a DNeasy PowerSoil kit (Qiagen, Hilden, Germany) and three individual tubes were pooled into one sample. The genomic DNAs were subjected to PCR using 341F and 805R primers to amplify the v3–v4 region of 16S rRNA gene for the predicted length of 428 bp, and adapters and indices were attached to the gene fragments by the library preparation procedures for the Illumina MiSeq sequencing. Paired-end sequencing (250 bp × 2) was performed, and the raw data of the DNA sequence (fastq) were analyzed using Mothur version 1.44.3 [24]. The data analysis was performed with a series of procedures including constructing contigs and quality controls such as removing sequencing errors, chimeric sequences using VSEARCH, and nonbacterial reads. Sequences were aligned using the Silva database (v. 132) [25], and clustered to operational taxonomic unit (OTU) via 3% dissimilarity. Taxa were assigned and classified using the RDP database [26].

#### 2.1.2. Experiment 2: Microaerobic Column Batch

To mimic a submerged rice paddy field, the same paddy soil (30 g) was added to the anammox medium (100 mL) contained in a 100-mL column type cylinder to let the oxygen gradient develop naturally along with the depth (Figure S1C). The soils were subsampled aseptically at the depths of 3 cm and 7 cm by using 10-mL serological pipettes for up to 80 days. For anammox activity, concentrations of ammonium and nitrite were analyzed along with the incubation times, and certain genes specific for nitrification and anammox were monitored via quantitative PCR analysis. Along with the *hzs*A gene, *amo*A and *nxr*B genes, encoding ammonium monooxygenase [27] and nitrite oxidoreductase [28], respectively, were monitored with incubation time for their absolute copy numbers. The primer sets for the *amo*A and *nxr*B genes are shown in Table S1.

#### 2.1.3. Experiment 3: Comparison of Aerobic and Anaerobic Incubations

To compare the development of anammox bacteria in the rice paddy soils between aerobic and anaerobic conditions, the same soils were incubated in the anammox media under aerobic and anaerobic conditions in two individual 1 L bottles (Figure S1D). Each 50 g of paddy soil was added to 90 mL of anammox medium; one culture was prepared aerobically, and the other culture was prepared anaerobically by purging with 100% N_2_. The sediment samples were collected from both the cultures at day 0 and day 30. Concentrations of nitrite and ammonia were analyzed to detect potential anammox reaction. Together with the *hzs*A gene, bacterial 16S rRNA gene was quantitatively analyzed for its absolute copy numbers to compare the ratios of anammox bacteria to total bacteria. The primer set of 16S rRNA gene (27F and 519R) is listed in Table S1. Also, to compare the aerobic and anaerobic enrichment incubations, bacterial community structures of both soils were analyzed via Illumina MiSeq sequencing of v3–v4 regions of 16S rRNA gene. The sequenced data were deposited at NCBI Sequence Read Archive (SRA) with the BioProject accession numbers of PRJNA1060457 and PRJNA1062134.

## 3. Results and Discussion

### 3.1. Anaerobic Batch Culture of the Rice Paddy Soil (Experiment 1)

**Figure 2:**
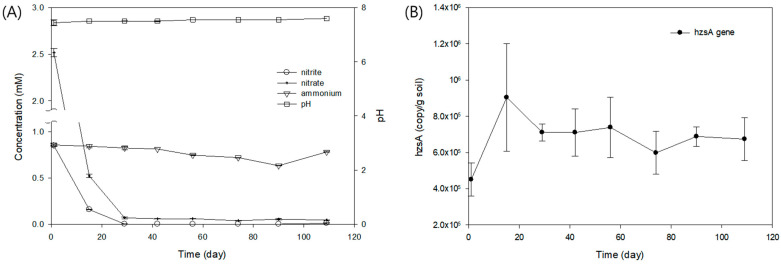
Anaerobic batch incubation. (**A**) Concentrations of nitrite, nitrate, and ammonium. (**B**) Absolute copy numbers of *hzs*A gene during the incubation period.

To examine whether anammox bacteria can be enriched from the paddy soil in anaerobically prepared batch culture, the soil was incubated anaerobically in the anammox medium for approximately 4 months. The pH of the soil batch was stable during the whole period of the incubation (Figure 2A). Concentrations of nitrite and nitrate decreased to almost below detection from the initial 0.85 mM and 2.5 mM, respectively after 29 days, and ammonia decreased gradually from the initial 0.86 mM to 0.63 mM by 90 days with a rebound up to 0.78 mM by 109 days (Figure 2A). The overall anammox reaction usually consumes ammonium and nitrite at a 1:1 ratio, resulting in dinitrogen [11]. However, in this incubation, nitrite consumption (0.85 mM) was three times larger than ammonium consumption (0.23 mM) (Figure 2A). This may not be only due to anammox reaction but possibly the denitrification reaction included as well in the soils/sediments. Probably the nitrate consumption (2.5 mM) indicated the presence of denitrification, which also reduces nitrite. The gene encoding hydrazine synthase subunit A, *hzs*A, which is one of the central enzymes for anammox reactions, was quantitated from the incubation soils via quantitative PCR (Figure 2B). The hydrazine synthase (HZS) combines ammonium (NH_4_^+^) with nitric oxide (NO) to generate hydrazine (N_2_H_4_), which is eventually oxidized to dinitrogen gas (N_2_) by hydrazine dehydrogenase [29]. The absolute copy number of the *hzs*A gene per gram of soil increased by nearly double to 9.0 × 10^5^ genes/g after 15 days of incubation from the initial 4.5 × 10^5^ genes/g and maintained approximately 7.0 × 10^5^ genes/g for the rest of the incubation period (Figure 2B). A decrease in the amounts of anammox-specific *hzs*A gene and nitrite used as an electron acceptor along with the gradual decrease in ammonia indicated potential enrichment of anammox bacteria from the anaerobic batch culture of the soil.

To compare the bacterial community changes in the anaerobic batch by time of culture, the 16S rRNA gene of the culture samples on days 1 (BR_D01), 15 (BR_D15), and 56 (BR_D56) was analyzed via MiSeq sequencing. At each time point two soil suspension samples (_a and _b) were prepared (a total of six samples), and genomic DNAs were extracted, followed by the gene library preparation for the Miseq sequencing. Data processing including contig assembly, quality controls, chimera removal using Mothur-moduled Vsearch [30], and nonbacterial sequence removal was performed by using Mothur, resulting in a reduction of sequence numbers from the initial 959,826 to the total final reads of 162,907 with the total OTU number of 13,497.

The smallest sequence number from the BR_D56_a was used for normalization of all the samples for alpha-diversity analysis (Table S2). The Good’s coverage values ranged from 0.90 to 0.93 among the six samples, suggesting that approximately more than 90% of the indigenous bacteria were sequenced. The OTU number and Chao1 index showed gradual decreases with the incubation time, though the differences were not large, indicating an overall decrease in species number along with time (Table S2). The diversity indices of Shannon and inverse Simpson showed rather complicated patterns of decreases at 15 days and then increases at 56 days (Table S2). Since these indices represent species evenness as well as species richness, some of dominant bacteria became relatively evenly enriched, less skewed with the incubation progressed.

**Figure 3:**
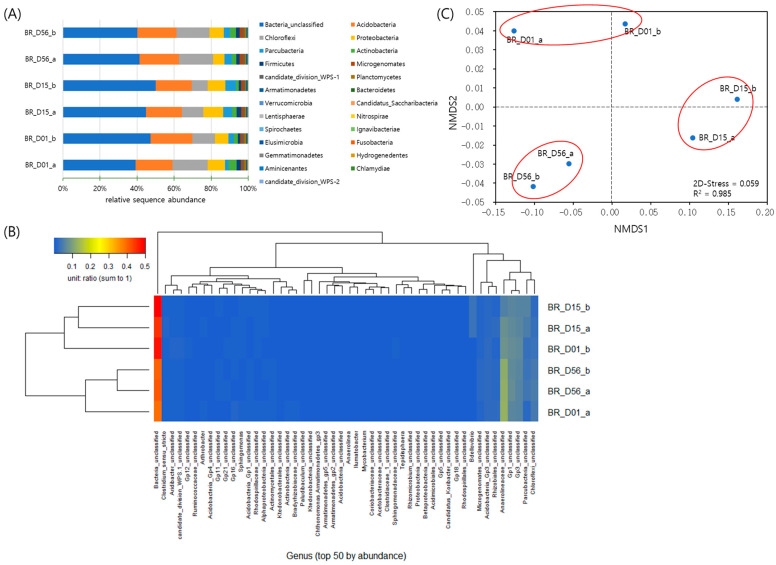
Anaerobic batch incubation. (**A**) Relative sequence abundance at phylum level over selected time points. (**B**) Sequence abundance-based heatmap with dendrograms using the Bray–Curtis dissimilarity. (**C**) Nonmetric multidimensional scaling (NMDS) of distances among the samples based on Yue and Clayton’s dissimilarity.

From the bar chart of relative sequence abundance, the dominant phyla *Acidobacteria*, *Chloroflexi*, *Proteobacteria*, *Parcubacteria*, *Actinobacteria*, and *Firmicutes* showed noticeable variations during the incubation time at the points of day 1, 15 days, and 56 days (Figure 3A and Figure S2). The communities of day 15 and day 56 were distinctively separated with distant groups via the dendrogram using Bray–Curtis dissimilarity in the heatmap (Figure S2), suggesting changes in the communities over time. The phylum *Planctomycetes* to which anammox bacteria belong appeared up to 0.5–0.9% of each sample.

The most abundant 50 genera among the total 337 were used for the sequence abundance-based heatmap with dendrograms using the Bray–Curtis dissimilarity (Figure 3B). A few genera including unclassified *Anaerolineaceae*, unclassified Gp1, unclassified Gp3, unclassified *Parcubacteria*, unclassified *Chloroflexi*, unclassified *Acidobacteria* Gp3, and unclassified *Rhizobiales* accounted for a large proportion of the bacterial communities across the samples (Figure 3B and Figure S3). As in the phylum level heatmap, the communities of day 15 and day 56 were grouped separately in the genus level heatmap (Figure 3B). In particular, the genus *Tepidisphaera* belonging to the phylum *Planctomycetes* was ranked 28th among 337 genera (Figure S3). Since little is known about this genus and its function with only one species [31], a potential linkage with the anammox reaction can be considered.

Distances among the samples were calculated based on the Yue and Clayton’s dissimilarity and analyzed for nonmetric multidimensional scaling (NMDS) (Figure 3C). The formed clusters of the two replicate samples at each time point indicate the changes in microbial communities over time.

### 3.2. Microaerobic Column Batch (Experiment 2)

In a sedimentary environment submerged in water such as paddy soil, there is a big difference in the dissolved oxygen concentration in the shallow (a few centimeters) and deep (tens of centimeters) sedimentary soils, and accordingly there is a big difference in the oxidation/reduction potential. Therefore, it can be expected that nitrifying bacteria that oxidize ammonia aerobically act in the shallow depth of sedimentary soil, and the nitrite (NO_2_^−^) generated from the nitrification reaction can be used as the electron acceptor for anammox bacteria, anaerobically oxidizing NH_4_^+^ at deeper soils. Since nitrite, which is essential for anammox reaction, exists in a transient state in the environment, nitrifying bacteria may induce anammox reaction.

**Figure 4:**
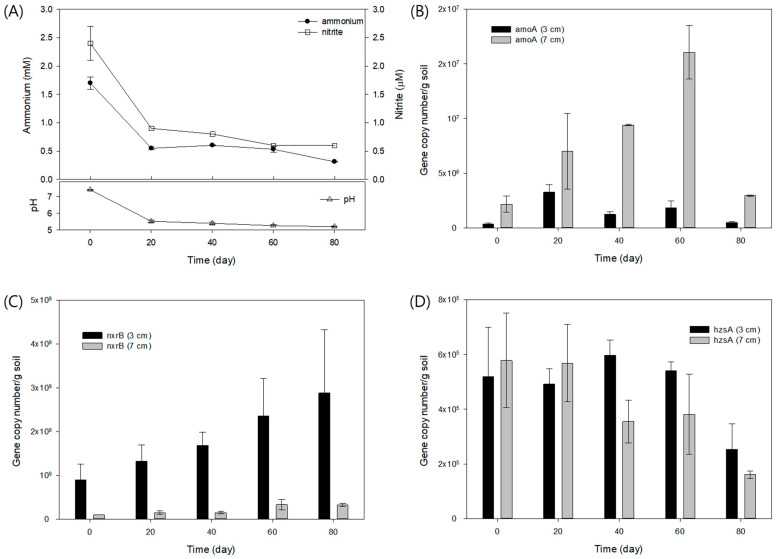
Microaerobic column batch incubation. (**A**) Chemistry data during the incubation period. (**B**–**D**) Absolute gene copy numbers of *amo*A, *nxr*B, and *hzs*A gene, respectively, at the sediment depths of 3 cm and 7 cm.

To mimic aqueous sedimentary environments such as streams and riverbeds, the previously anammox-enriched paddy soils were submerged in the column-type of freshwater media with natural oxygen gradient over the depth (Figure S1C). The pH, nitrite (NO_2_^−^), and ammonium (NH_4_^+^) were monitored over time (Figure 4A). A decrease in pH was observed as the culture progressed, which was presumed to be due to the production of NO_2_^−^ and NO_3_^−^ via nitrification and/or oxidation of NH_4_^+^ provided in the culture medium at the initial concentration of 0.5 mM. Nitrite concentration decreased along with ammonia, which may be the result of denitrification and/or anammox reaction (Figure 4A). Specific genes mediating the reactions of nitrification and anammox were detected over time and along with the depths (Figure 4B–D). Nitrification is characterized mostly by two-step reactions using *amo*A gene encoding ammonia monooxygenase subunit A, which oxidizes ammonia to nitrite, and *nxr*B gene encoding the beta subunit of nitrite oxidoreductase, which oxidizes nitrite to nitrate. Absolute gene copy number of *amo*A in the unit soil increased at a depth of 3 cm only in the early period (20 days), whereas it increased gradually toward the latter part of the reaction (60 days) at a depth of 7 cm (Figure 4B). This might suggest that ammonia oxidation to nitrite increased in the sediment more actively in the deeper sediment at 7 cm, which was consistent with the decrease in ammonia in the media (Figure 4A). Similarly, the *nxr*B gene also showed a gradual increase in the sediment, but more actively in the upper parts at 3 cm, indicating oxidation of nitrite to nitrate, concomitantly with oxidation of ammonia (Figure 4C). On the contrary, at the depth of 7 cm, there was not much increase in *nxr*B, which may suggest nitrite utilization not by aerobic nitrification, but potentially by anaerobic reaction of anammox. The *hzs*A gene, which is a subunit of the hydrazine synthase, representing a unique phylogenetic marker for anammox bacteria, was found to exist across the depths, despite relatively low absolute amounts in both the depths compared to *amo*A and *nxr*B genes (Figure 4D).

### 3.3. Comparison of Aerobic and Anaerobic Incubations (Experiment 3)

**Figure 5:**
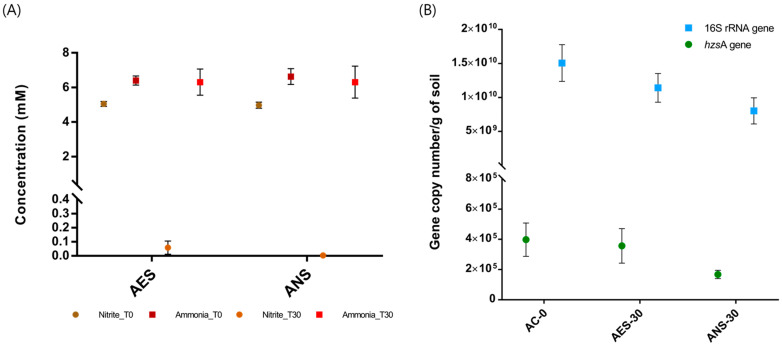
Comparison of short-term incubations. (**A**) Concentrations of nitrite and ammonium, and (**B**) absolute gene copy numbers of 16S rRNA gene and *hzs*A gene from the compared aerobic and anaerobic incubations for selected times.

To compare the development of anammox bacteria in the rice paddy soils between aerobic and anaerobic conditions, the same soils were incubated in the anammox media under aerobic and anaerobic conditions. It was found that the initial amounts of nitrite were mostly consumed by 30 days in both the aerobic (AES) and anaerobic (ANS) incubations (Figure 5A), whereas ammonium decreases were minor compared to the nitrite decreases in both the conditions. This may suggest that denitrification was dominant rather than nitrification and/or anammox in both the AES and ANS incubations.

From the same incubations, bacterial 16S rRNA gene and *hzs*A gene were quantitatively analyzed. The initial number of 16S rRNA gene (1.51 × 10^10^ ± 2.70 × 10^9^ copy/g soil) decreased to 1.14 × 10^10^ ± 2.11 × 10^9^ copy/g soil and 8.02 × 10^9^ ± 1.92 × 10^9^ copy/g both in the AES and ANS, respectively, after 30 days of incubation (Figure 5B). The *hzs*A gene also showed a similar tendency to the 16S rRNA gene, and the gene copy numbers were reduced from the initial number of 3.98 × 10^5^ ± 1.10 × 10^5^ copy/g soil to 3.57 × 10^5^ ± 1.14 × 10^5^ copy/g soil and 1.68 × 10^5^ ± 2.75 × 10^4^ copy/g soil, respectively, for the AES and ANS at 30 days (Figure 5B). Although the absolute amounts of the genes decreased over time, the ratios of *hzs*A gene to 16S rRNA gene increased over time, from the initial 4.02 to 5.95 and 5.51, respectively, for the AES and ANS (Table S3). The decrease in bacterial abundances inferred by 16S rRNA gene copies was probably due to the incubation conditions without continuous feeding or added organic materials. However, as the incubations progressed, the ratios of *hzs*A gene increased, indicating that conditions were changing favorably for anammox rather than many types of heterotrophs. The aerobic incubation, AES showed a higher ratio of *hzs*A gene rather than the anaerobic incubation, ANS, which may suggest that the gradual consumption of dissolved oxygen may have developed a microaerobic or oxic-anoxic transition zone, probably favored by anammox, in the AES incubation. The oxygen in the initial atmosphere and the medium of the AES incubation may have been consumed over time, and a microaerobic environment would have been created as it progressed. Although it was difficult to estimate the anammox response using only the chemistry data of nitrite, ammonium, etc., the presence of anammox bacteria could be estimated to some extent due to the relative increase in the anammox-specific gene. Also, the chemistry data could be explained by combined reactions of anammox and (de)nitrification, specifically at the redox boundary.

The bacterial community was analyzed for the initial soil (ACT00), aerobic soil at 30 days (AES30), and anaerobic soil at 30 days (ANS30) by amplifying bacterial 16S rRNA gene v4 region using the Illumina MiSeq System. The final sequence read was 37,384 for the whole three samples, and the total number of OTUs was 4442. Looking at the diversity indices of bacterial communities, the observed species (or OTU) and Chao1 index, indicating the species richness or number of species, showed a larger decrease in the aerobic soil than the anaerobic soil (Table S4). Shannon and inverse Simpson indices, indicating both species abundance and evenness, decreased similarly with the OTU numbers and Chao1 index.

The whole bacterial community across the samples was classified into 24 phyla (Figure S4A). The major phyla included *Chloroflexi*, *Acidobacteria*, *Proteobacteria*, *Firmicutes*, etc., (Figure S4B) and phylum *Planctomycetes*, to which anammox bacteria belong, occupied approximately 2% of the sequence abundances (2.1%, 1.7%, and 1.9%, respectively, for ACT00, AES30, and ANS30). Phyla *Chloroflexi* and *Proteobacteria* increased rather in the aerobic incubation (AES30) than ANS30, and in the phylum *Chloroflexi,* there are many types of phototrophs, possibly suggesting paddy soil conditions.

The bacterial communities indicated a total of 268 genera, with 20 genera showing ratios of 1% or more in individual samples (Figure S5). Two genera of unclassified *Bacteria* and unclassified *Anaerolineaceae* occupied approximately 41–42% of each sample. The family *Anaerolineaceae* was reported in aquatic sediments and anaerobic digesters as fermenters [32], which may be characteristic for long-term anaerobic incubation. There was no significant difference in taxa between the samples at genus level. Among phylum *Plactomycetes*, a taxon, classified as *Planctomycetaceae*_unclassified which was not classified below the family, showed the highest sequence abundance ratios at genus level (0.66%, 0.77%, and 0.54%, respectively, for ACT00, AES30, and ANS30). A total of nine genera of *Planctomycetes* were identified, including three taxa not classified below certain levels: *Phycisphaerae*_unclassified, *Phycisphaera*, *Tepidisphaera*, *Planctomycetes*_unclassified, *Aquisphaera*, *Gemmata*, *Pirellula*, *Planctomycetaceae*_unclassified, and *Zavarzinella*. However, no known anammox bacterium was identified from the sequences.

Analyzing the time-series chemistry data of the microaerobic column incubation, the *nxr*B gene, which encodes the beta-subunit of nitrite oxidoreductase, showed an increase over time compared to the *amo*A gene at a depth of 3 cm (Figure 4B,C). Additionally, at this depth, the *hzs*A gene remained relatively stable compared to its levels at a depth of 7 cm (Figure 4D). The anammox reaction requires nitrite as the electron acceptor, with nitrite being reduced to nitric oxide via nitrate reductase (NirS) in the anammox process [33]. However, nitrite reduction can also occur via nitrite oxidoreductase [28]. Therefore, the simultaneous increase and/or stable levels of the *nxr*B and *hzs*A genes at the 3 cm depth may strongly indicate anammox activity.

Based on these results, anammox activity was more prominent in the microaerobic depth than in the deeper anaerobic conditions. This could be because nitrite is more readily available in microaerobic conditions due to the activity of ammonia-oxidizing bacteria, such as the aerobic nitrifier *Nitrosomonas*, whereas nitrification does not occur under anaerobic conditions, where anammox bacteria compete for nitrite with denitrifying bacteria. Studies have shown that the combination of partial nitrification and anammox creates optimal conditions for anammox bacteria growth, facilitating enhanced nitrogen removal from municipal wastewater [34,35]. In such redox (anoxic/oxic) transition zones, the presence of substrates like nitrite can support the growth of anammox bacteria.

In Experiment 1, nitrite concentration was nearly depleted after one month, coinciding with an increase in the anammox-specific gene *hzs*A (Figure 2). We speculated that anammox activity would manifest within one month and thus compared aerobic and anaerobic incubations over this time frame in Experiment 3. Although the microbial communities were not directly comparable due to different sampling times, there were shared abundances of certain taxa at the phylum level, including *Chloroflexi*, *Acidobacteria*, *Parcubacteria*, and *Actinobacteria*, etc. (Figure 3 and Figure S4). Furthermore, both 16S amplicon sequencing analyses detected the phylum *Planctomycetes*, suggesting the potential presence of anammox microorganisms.

## 4. Conclusions

Although we could not confirm the anammox reaction with chemistry data, based on the chemistry data and the bacterial specific functional genes, we could speculate reactions of not only anammox bacteria but also denitrifying bacteria and nitrifying bacteria in the paddy soils. It might be difficult to completely rule out the anammox reaction with only physicochemical data. The increase in the ratio of the hydrazine synthetase gene compared to the 16S rRNA gene may suggest evidence for an active but minor reaction of anammox bacteria in the paddy soils. Also, there may be the potential for anammox bacteria presence in the unclassified *Planctomycetes* identified in the bacterial communities.

## Data Availability

Raw sequence data generated for this study are available at the NCBI Sequence Read Archive (SRA) with the BioProject accession numbers of PRJNA1060457 and PRJNA1062134.

## Supplementary Materials

The following supporting information can be downloaded at: https://www.mdpi.com/article/10.3390/biology13070548/s1, Table S1. Primer sequences used in this study; Table S2. Alpha-diversity of the microbial communities from the anaerobic batch incubation (Experiment 1); Table S3. Ratios of *hzs*A gene to 16S rRNA gene from the compared aerobic and anaerobic incubations (Experiment 3); Table S4. Alpha-diversity of the microbial communities from the compared aerobic and anaerobic incubations (Experiment 3); Figure S1. Anammox enrichment batches using rice paddy soils from Rural Development Administration field, Jeonju, South Korea (A). (B) Experiment 1—anaerobic incubation, (C) Experiment 2—microaerobic column incubation, (D) Experiment 3—compared aerobic and anaerobic incubations; Figure S2. Heatmap of the sequence abundance at the phylum level for the anaerobic incubation (Experiment 1). Vertical and horizontal dendrograms based on the Bray–Curtis dissimilarity; Figure S3. Relative sequence abundance of the top 30 genera for the anaerobic incubation (Experiment 1); Figure S4. Relative sequence abundance (A) and its heatmap (B) of phyla from the compared aerobic and anaerobic incubations (Experiment 3); Figure S5. Relative sequence abundance (A) and its heatmap (B) of top 20 genera from the compared aerobic and anaerobic incubations (Experiment 3). References [36,37,38,39,40] are cited in the Supplementary Materials.

## References

[1] Ahmed M., Rauf M., Mukhtar Z., Saeed N.A. (2017). Excessive use of nitrogenous fertilizers: An unawareness causing serious threats to environment and human health. Environ. Sci. Pollut. Res..

[2] Ibrahim K.A., Naz M.Y., Shukrullah S., Sulaiman S.A., Ghaffar A., AbdEl-Salam N.M. (2020). Nitrogen pollution impact and remediation through low cost starch based biodegradable polymers. Sci. Rep..

[3] Liu M., Shang F., Lu X., Huang X., Song Y., Liu B., Zhang Q., Liu X., Cao J., Xu T. (2022). Unexpected response of nitrogen deposition to nitrogen oxide controls and implications for land carbon sink. Nat. Commun..

[4] Mosley O.E., Gios E., Close M., Weaver L., Daughney C., Handley K.M. (2022). Nitrogen cycling and microbial cooperation in the terrestrial subsurface. ISME J..

[5] Jia H., Yuan Q. (2016). Removal of nitrogen from wastewater using microalgae and microalgae–bacteria consortia. Cogent Environ. Sci..

[6] Rajta A., Bhatia R., Setia H., Pathania P. (2020). Role of heterotrophic aerobic denitrifying bacteria in nitrate removal from wastewater. J. Appl. Microbiol..

[7] Zhou Y., Zhu Y., Zhu J., Li C., Chen G. (2023). A comprehensive review on wastewater nitrogen removal and its recovery processes. Int. J. Environ. Res. Public Health.

[8] Kraft B., Tegetmeyer H.E., Sharma R., Klotz M.G., Ferdelman T.G., Hettich R.L., Geelhoed J.S., Strous M. (2014). The environmental controls that govern the end product of bacterial nitrate respiration. Science.

[9] Kuypers M.M.M., Marchant H.K., Kartal B. (2018). The microbial nitrogen-cycling network. Nat. Rev. Microbiol..

[10] Seruga P., Krzywonos M., Pyżanowska J., Urbanowska A., Pawlak-Kruczek H., Niedźwiecki Ł. (2019). Removal of ammonia from the municipal waste treatment effluents using natural minerals. Molecules.

[11] van de Graaf A.A., de Bruijn P., Robertson L.A., Jetten M.S.M., Kuenen J.G. (1996). Autotrophic growth of anaerobic ammonium-oxidizing micro-organisms in a fluidized bed reactor. Microbiology.

[12] Date Y., Isaka K., Ikuta H., Sumino T., Kaneko N., Yoshie S., Tsuneda S., Inamori Y. (2009). Microbial diversity of anammox bacteria enriched from different types of seed sludge in an anaerobic continuous-feeding cultivation reactor. J. Biosci. Bioeng..

[13] Du R., Horn H., Cao S. (2023). Maximizing anammox in mainstream wastewater treatment: An integrated nitrite producing approach. Chem. Eng. J..

[14] Fan N.-S., Bai Y.-H., Wu J., Zhang Q., Fu J.-J., Zhou W.-L., Huang B.-C., Jin R.-C. (2020). A two-stage anammox process for the advanced treatment of high-strength ammonium wastewater: Microbial community and nitrogen transformation. J. Clean. Prod..

[15] Fu Y., Wen X., Huang J., Sun D., Jin L. (2023). Advances in the efficient enrichment of anammox bacteria. Water.

[16] Kuenen J.G. (2008). Anammox bacteria: From discovery to application. Nat. Rev. Microbiol..

[17] Niederdorfer R., Hausherr D., Palomo A., Wei J., Magyar P., Smets B.F., Joss A., Bürgmann H. (2021). Temperature modulates stress response in mainstream anammox reactors. Commun. Biol..

[18] Liu L., Hu M., Wang C., Qi W., Peng Y. (2023). Enrichment of anammox bacteria using anammox sludge as a primer combined with ordinary activated sludge. Sustainability.

[19] Ren Z.-Q., Wang H., Zhang L.-G., Du X.-N., Huang B.-C., Jin R.-C. (2022). A review of anammox-based nitrogen removal technology: From microbial diversity to engineering applications. Bioresour. Technol..

[20] Lu Y., Natarajan G., Nguyen T.Q.N., Thi S.S., Arumugam K., Seviour T., Williams R.B.H., Wuertz S., Law Y. (2022). Controlling anammox speciation and biofilm attachment strategy using N-biotransformation intermediates and organic carbon levels. Sci. Rep..

[21] Khanal A., Lee J.-H. (2020). Functional diversity and abundance of nitrogen cycle-related genes in paddy soil. Appl. Biol. Chem..

[22] Khanal A., Lee S., Lee J.-H. (2020). Detection and potential abundances of anammox bacteria in the paddy soil. Korean J. Environ. Agric..

[23] Kartal B., Keltjens J.T. (2016). Anammox biochemistry: A tale of heme c proteins. Trends Biochem. Sci..

[24] Schloss P.D., Westcott S.L., Ryabin T., Hall J.R., Hartmann M., Hollister E.B., Lesniewski R.A., Oakley B.B., Parks D.H., Robinson C.J. (2009). Introducing mothur: Open-source, platform-independent, community-supported software for describing and comparing microbial communities. Appl. Environ. Microbiol..

[25] Quast C., Pruesse E., Yilmaz P., Gerken J., Schweer T., Yarza P., Peplies J., Glockner F.O. (2013). The SILVA ribosomal RNA gene database project: Improved data processing and web-based tools. Nucleic Acids Res..

[26] Wang Q., Garrity G.M., Tiedje J.M., Cole J.R. (2007). Naïve Bayesian classifier for rapid assignment of rRNA sequences into the new bacterial taxonomy. Appl. Environ. Microbiol..

[27] Hatzenpichler R. (2012). Diversity, physiology, and niche differentiation of ammonia-oxidizing archaea. Appl. Environ. Microbiol..

[28] Pester M., Maixner F., Berry D., Rattei T., Koch H., Lücker S., Nowka B., Richter A., Spieck E., Lebedeva E. (2014). NxrB encoding the beta subunit of nitrite oxidoreductase as functional and phylogenetic marker for nitrite-oxidizing Nitrospira. Environ. Microbiol..

[29] Wang Y., Ma X., Zhou S., Lin X., Ma B., Park H.-D., Yan Y. (2016). Expression of the *nir*S, *hzs*A, and *hdh* genes in response to nitrite shock and recovery in *Candidatus* Kuenenia stuttgartiensis. Environ. Sci. Technol..

[30] Rognes T., Flouri T., Nichols B., Quince C., Mahé F. (2016). VSEARCH: A versatile open source tool for metagenomics. PeerJ.

[31] Kovaleva O.L., Merkel A.Y., Novikov A.A., Baslerov R.V., Toshchakov S.V., Bonch-Osmolovskaya E.A. (2015). *Tepidisphaera mucosa* gen. nov., sp. nov., a moderately thermophilic member of the class *Phycisphaerae* in the phylum *Planctomycetes*, and proposal of a new family, *Tepidisphaeraceae* fam. nov., and a new order, *Tepidisphaerales* ord. nov. Int. J. Syst. Evol. Microbiol..

[32] McIlroy S.J., Kirkegaard R.H., Dueholm M.S., Fernando E., Karst S.M., Albertsen M., Nielsen P.H. (2017). Culture-independent analyses reveal novel *Anaerolineaceae* as abundant primary fermenters in anaerobic digesters treating waste activated sludge. Front. Microbiol..

[33] van Niftrik L., Geerts W.J.C., van Donselaar E.G., Humbel B.M., Webb R.I., Fuerst J.A., Verkleij A.J., Jetten M.S.M., Strous M. (2008). Linking ultrastructure and function in four genera of anaerobic ammonium-oxidizing bacteria: Cell plan, glycogen storage, and localization of cytochrome *c* proteins. J. Bacteriol..

[34] Chen J., Zhang X., Zhang X., Zhu Z., Wu Y., Wang C., Cai T., Li X., Wu P. (2022). Mainstream anammox driven by micro-oxygen nitrification and partial denitrification using step-feed for advanced nitrogen removal from municipal wastewater. J. Clean. Prod..

[35] Hou X.H., Li X.Y., Zhu X.R., Li W.Y., Kao C.K., Peng Y.Z. (2024). Advanced nitrogen removal from municipal wastewater through partial nitrification-denitrification coupled with anammox in step-feed continuous system. Bioresour. Technol..

[36] Brunk C.F., Avaniss-Aghajani E., Brunk C.A. (1996). A computer analysis of primer and probe hybridization potential with bacterial small-subunit rRNA sequences. Appl. Environ. Microbiol..

[37] Harhangi H.R., Le Roy M., van Alen T., Hu B.L., Groen J., Kartal B., Tringe S.G., Quan Z.X., Jetten M.S., Op den Camp H.J. (2012). Hydrazine synthase, a unique phylomarker with which to study the presence and biodiversity of anammox bacteria. Appl. Environ. Microbiol..

[38] Herlemann D.P.R., Labrenz M., Jürgens K., Bertilsson S., Waniek J.J., Andersson A.F. (2011). Transitions in bacterial communities along the 2000 km salinity gradient of the Baltic Sea. ISME J..

[39] Lücker S., Wagner M., Maixner F., Pelletier E., Koch H., Vacherie B., Rattei T., Damsté J.S.S., Spieck E., Le Paslier D. (2010). A *Nitrospira* metagenome illuminates the physiology and evolution of globally important nitrite-oxidizing bacteria. Proc. Natl. Acad. Sci. USA.

[40] Rotthauwe J.H., Witzel K.P., Liesack W. (1997). The ammonia monooxygenase structural gene *amo*A as a functional marker: Molecular fine-scale analysis of natural ammonia-oxidizing populations. Appl. Environ. Microbiol..

