# Supplementary material for: Evidence of Potential Anammox Activities from Rice Paddy Soils in Microaerobic and Anaerobic Conditions
Source: Biology (Basel). 2024 Jul 19;13(7):548. doi: 10.3390/biology13070548 (PMC11273733; doi:10.3390/biology13070548)
Supplement: Supplementary file 1 [file biology-13-00548-s001.zip › biology-3079819-supplementary.pdf]

Supplementary Material

Evidence of Potential Anammox Activities from Rice Paddy Soils in Microaerobic and Anaerobic Conditions

Anamika Khanal, Hyung-Geun Song, Yu-Sung Cho, Seo-Yeon Yang, Won-Seok Kim, Alpana Joshi, Jiho Min, Ji-Hoon Lee

List of contents

Table S1.

Table S2.

Table S3.

Table S4.

Figure S1.

Figure S2.

Figure S3.

Figure S4.

Figure S5.

Table S1. Primer sequences used in this study

Primers	Sequence 5'- 3'	Annealing temperature (°C)	References
27F	AGAGTTTGATCMTGGCTCAG	55°C	Brunk et al. (1996)
519R	GWATTACCGCGGCKGCTG	55°C	Brunk et al. (1996)
341F	CCTACGGGNGGCWGCAG	used in MiSeq	Herlemann et al. (2011)
805R	GACTACHVGGGTATCTAATCC	used in MiSeq	Herlemann et al. (2011)
amoA-1F	GGGGTTTCTACTGGTGGT	52°C	Rotthauwe et al. (1997)
amoA-2R	CCCCTCKGSAAAGCCTTC	52°C	Rotthauwe et al. (1997)
nxrB169f	TACATGTGGTGGAACA	42°C	Lücker et al. (2010)
nxrB638r	CGGTTCTGGTCRATCA	42°C	Lücker et al. (2010)
hzsA_382F	GGYGGDTGYCAGATATGGG	59°C	Harhangi et al. (2012)
hzsA_1857R	AAABGGYGAATCATARTGGC	59°C	Harhangi et al. (2012)

Table S2. Alpha-diversity of the microbial communities from the anaerobic batch incubation (Experiment 1)

Sample	normalized sequences	coverage	species observed (OTU)	Chao1	Shannon	inverse Simpson
BR_D01_a	20159	0.9029 ±0.0013	3728.0±19.8	6374.4 ±125.0	6.681±0.007	168.1±2.1
BR_D01_b	20159	0.9110 ±0.0015	3310.9±24.0	6091.3 ±165.0	6.333±0.011	97.3±1.7
BR_D15_a	20159	0.9188 ±0.0012	3289.5±17.6	5317.6 ±105.1	6.341±0.008	93.3±1.2
BR_D15_b	20159	0.9190 ±0.0013	3170.7±21.3	5235.9 ±119.5	6.122±0.010	72.9±1.0
BR_D56_a	20159	0.9334	3083.0	4345.1	6.448	131.6
BR_D56_b	20159	0.9208 ±0.0011	3299.9±17.4	5121.7 ±94.1	6.548±0.007	159.1±2.0

Table S3. Ratios of *hzs*A gene to 16S rRNA gene from the compared aerobic and anaerobic incubations (Experiment 3)

Sample	The ratio of *hzs*A to 16S rRNA gene (by mean)
Initial (AC-0)	4.019
Aerobic, after 30 d (AES-30)	5.949
Anaerobic, after 30 d (ANS-30)	5.510

Table S4. Alpha-diversity of the microbial communities from the compared aerobic and anaerobic incubations (Experiment 3)

Sample	No. of sequence	Good's coverage	observed species (OTU)	Chao1	Shannon	inverse Simpson
AC_T00	12402	0.8868±0.0003	2588.2±2.7	5089±28	6.632±0.001	237.4±0.5
AES_30	12402	0.9063	2161	4344	6.364	194.0
ANS_30	12402	0.8988±0.0003	2303.8±3.0	4841±36	6.427±0.002	187.5±0.5

Figure S1. Anammox enrichment batches using the rice paddy soils from Rural Development Administration field, Jeonju, South Korea (A). (B) Experiment 1 – Anaerobic incubation, (C) Experiment 2 – Microaerobic column incubation, (D) Experiment 3 – Compared aerobic and anaerobic incubations.

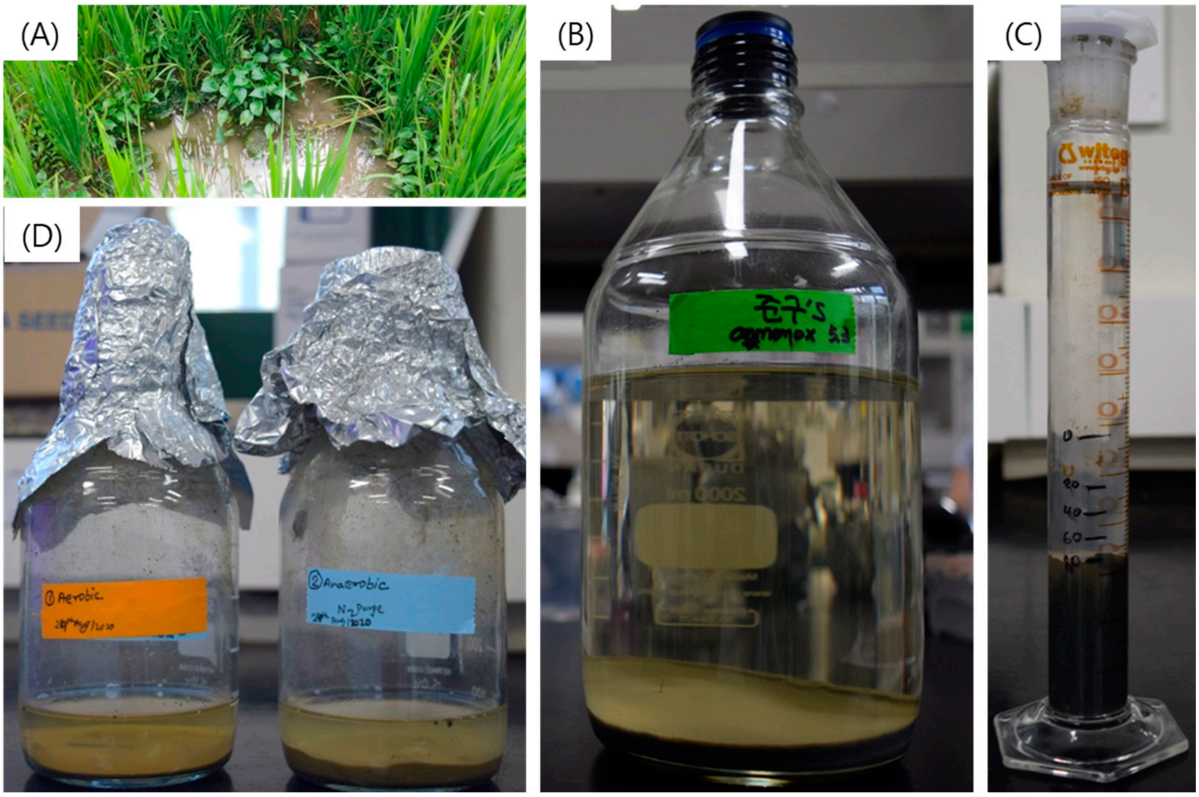

Figure S2. Heatmap of the sequence abundance at the phylum level for the anaerobic incubation (Experiment 1). Vertical and horizontal dendrograms based on the Bray-Curtis dissimilarity.

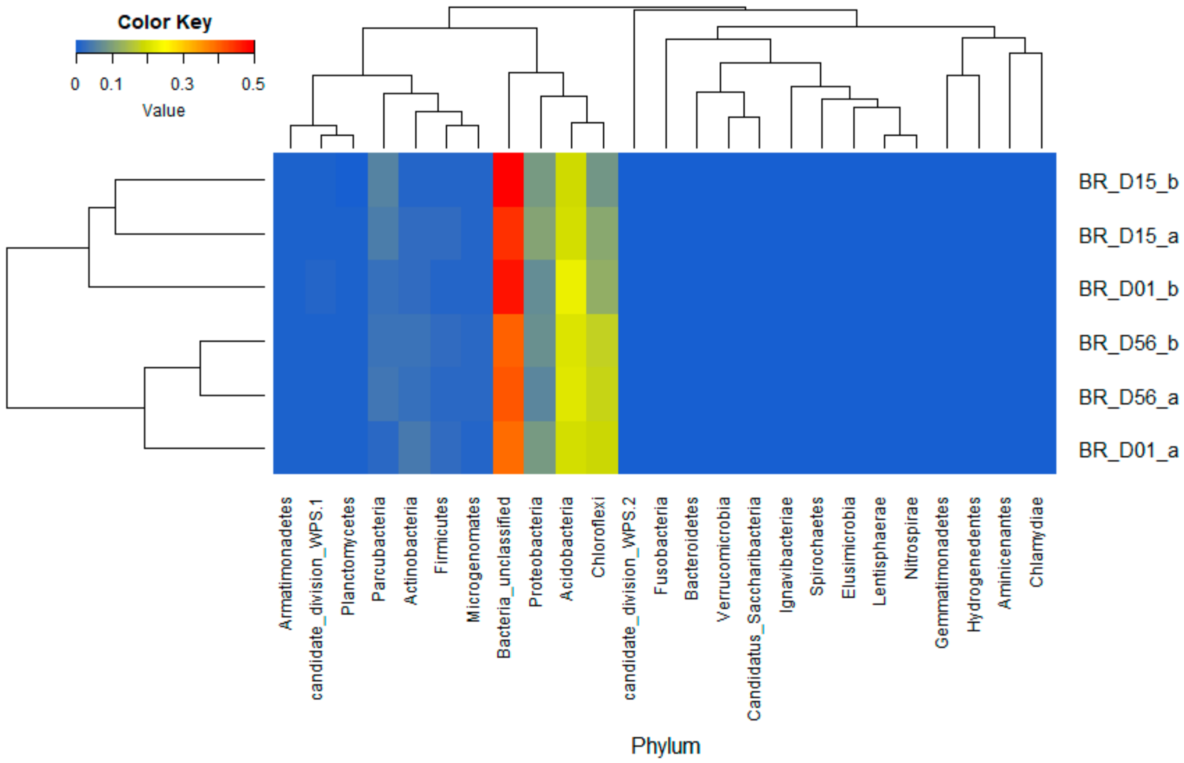

Figure S3. Relative sequence abundance of the top 30 genera for the anaerobic incubation (Experiment 1).

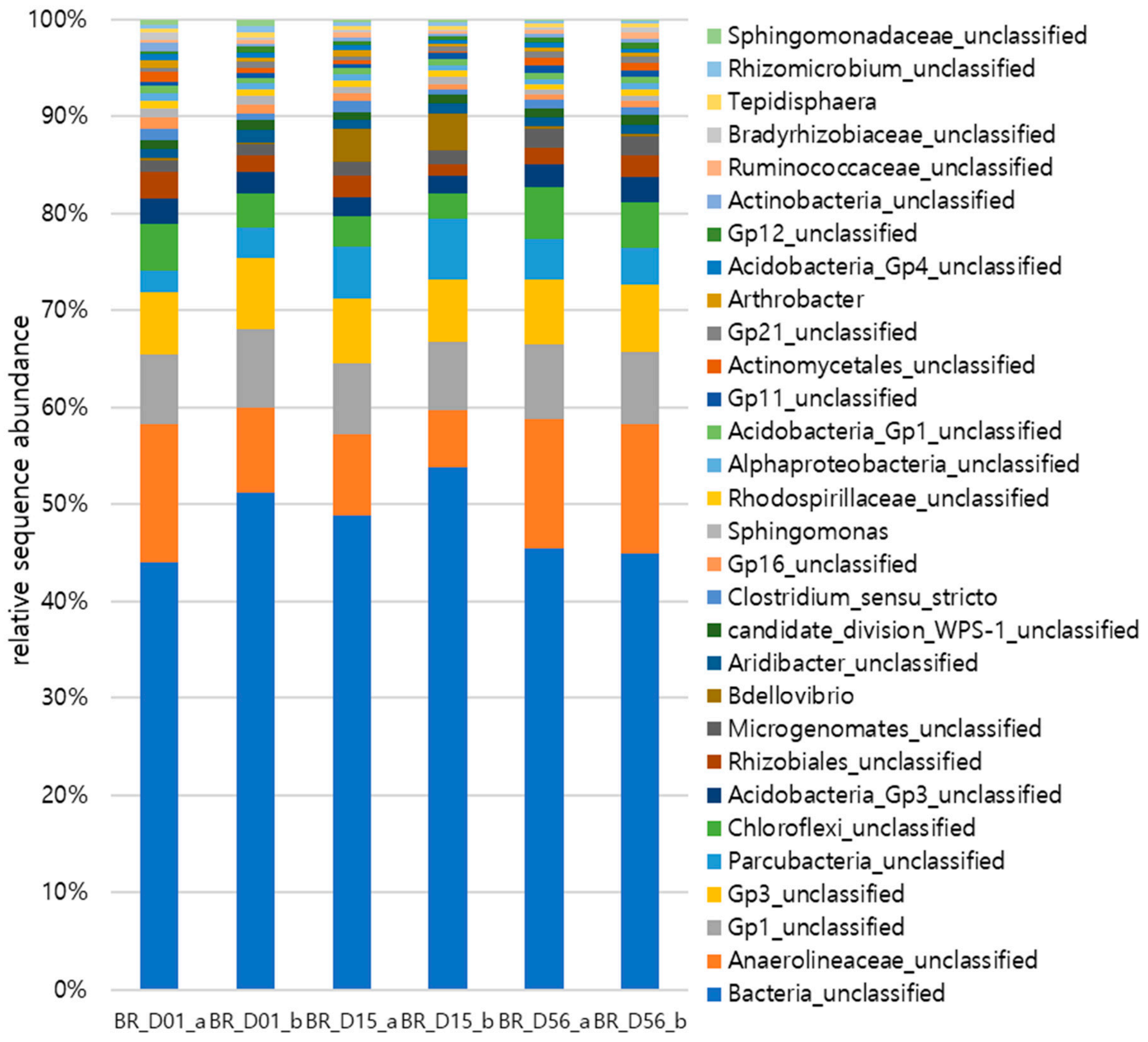

Figure S4. Relative sequence abundance (A) and its heatmap (B) of phyla from the compared aerobic and anaerobic incubations (Experiment 3).

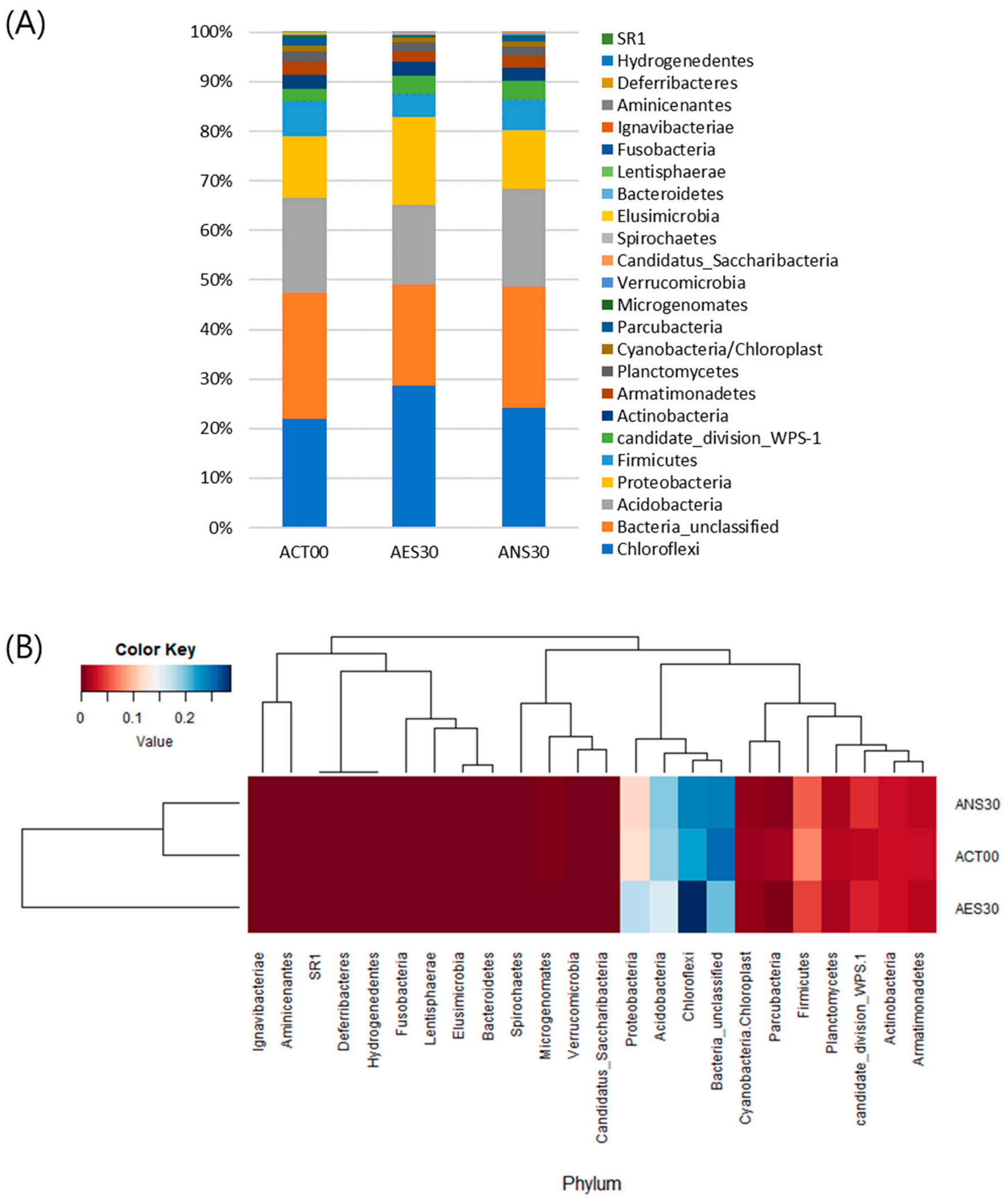

Figure S5. Relative sequence abundance (A) and its heatmap (B) of top 20 genera from the compared aerobic and anaerobic incubations (Experiment 3).

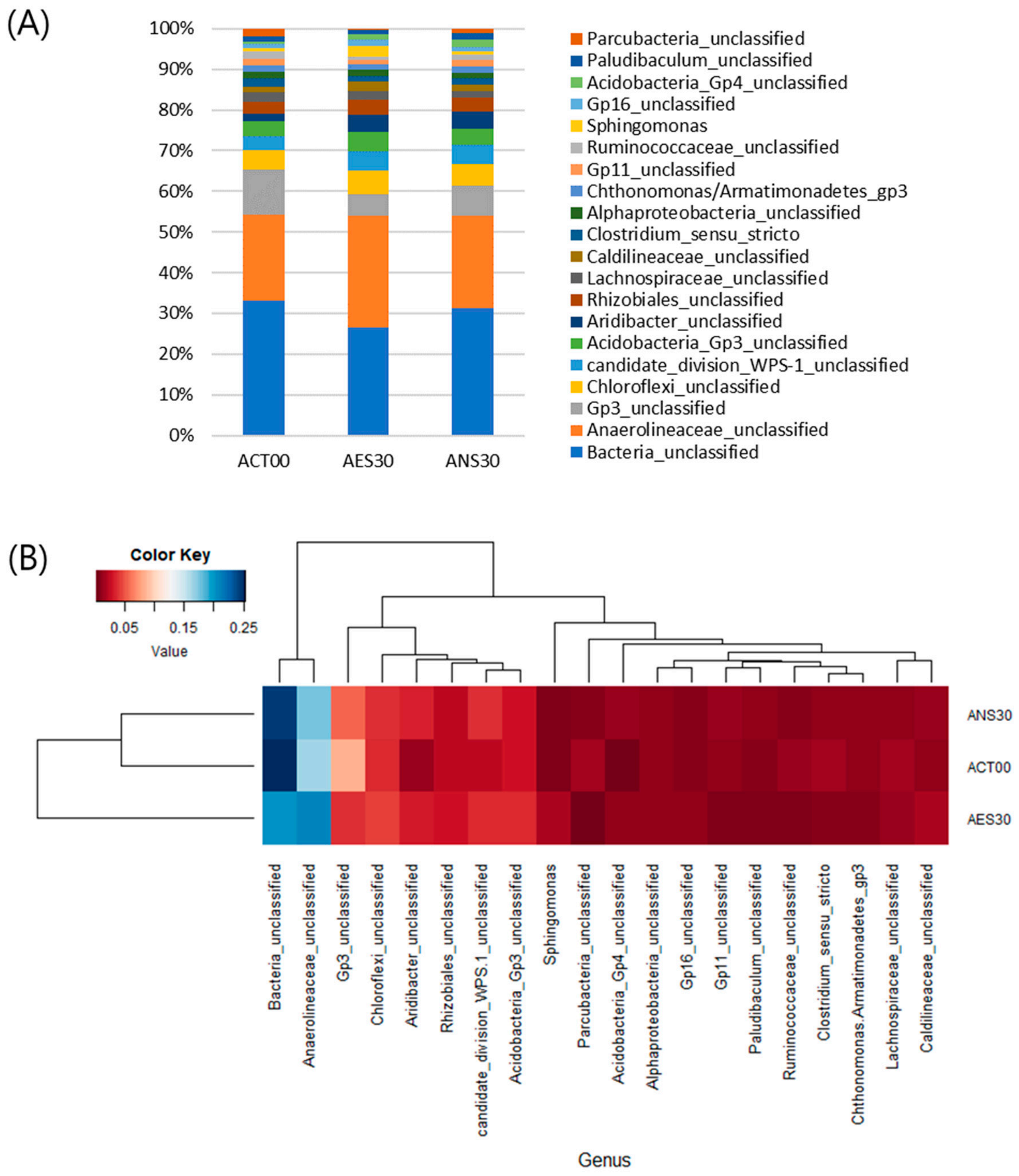

References

Brunk, C.F.; Avaniss-Aghajani, E.; Brunk, C.A. A computer analysis of primer and probe hybridization potential with bacterial small-subunit rRNA sequences. *Appl. Environ. Microbiol.* **1996**, *62*, 872–879.

Harhangi, H.R.; Le Roy, M.; van Alen, T.; Hu, B.L.; Groen, J.; Kartal, B.; Tringe, S.G.; Quan, Z.X.; Jetten, M.S.; Op den Camp, H.J. Hydrazine synthase, a unique phylomarker with which to study the presence and biodiversity of anammox bacteria. *Appl. Environ. Microbiol.* **2012**, *78*, 752–758.

Herlemann, D.P.R.; Labrenz, M.; Jürgens, K.; Bertilsson, S.; Waniek, J.J.; Andersson, A.F. Transitions in bacterial communities along the 2000 km salinity gradient of the Baltic Sea. *ISME J.* **2011**, *5*, 1571–1579.

Lücker, S.; Wagner, M.; Maixner, F.; Pelletier, E.; Koch, H.; Vacherie, B.; Rattei, T.; Damsté, J.S.S.; Spieck, E.; Le Paslier, D.; et al. A *Nitrospira* metagenome illuminates the physiology and evolution of globally important nitrite-oxidizing bacteria. *Proc. Natl. Acad. Sci. USA* **2010**, *107*, 13479–13484.

Rotthauwe, J.H.; Witzel, K.P.; Liesack, W. The ammonia monooxygenase structural gene *amo*A as a functional marker: molecular fine-scale analysis of natural ammonia-oxidizing populations. *Appl. Environ. Microbiol.* **1997**, *63*, 4704–4712.
